# **RETRACTION:** Melatonin Modulates Mitophagy, Innate Immunity and Circadian Clocks in a Model of Viral‐Induced Fulminant Hepatic Failure

**DOI:** 10.1111/jcmm.70983

**Published:** 2025-12-09

## Abstract

**RETRACTION**: CrespoI. , Fernández‐PalancaP. , San‐MiguelB. , ÁlvarezM. , González‐GallegoJ. , and TuñónM.J. , “Melatonin Modulates Mitophagy, Innate Immunity and Circadian Clocks in a Model of Viral‐Induced Fulminant Hepatic Failure,” Journal of Cellular and Molecular Medicine 24, no. 13 (2020): 7625–7636, 10.1111/jcmm.15398.32468679 PMC7339179

The above article, published online on 29 May 2020 in Wiley Online Library (http://onlinelibrary.wiley.com/), has been retracted by agreement between the journal Editor‐in‐Chief, Stefan N. Constantinescu; the Foundation for Cellular and Molecular Medicine; and John Wiley & Sons Ltd. Following publication, concerns were raised by third parties regarding duplications in panels C and F in Figure 1B. Additional concerns of duplication and splicing were also raised for Figures 2C and 3B, which were investigated and confirmed by the publisher; additionally, portions of Figure 4 were duplicated from Figure 3 of an earlier article by some of the same authors (González‐Fernández et al. 2018 [https://doi.org/10.3389/fphar.2018.00556]). The authors provided their raw data, but this was not sufficient to resolve the concerns, and the authors were unable to provide a satisfactory explanation. The retraction has been agreed upon because of concerns that portions of the figures were duplicated, affecting the interpretation of the data and results presented.

**RETRACTION**: I. Crespo , P. Fernández‐Palanca , B. San‐Miguel , M. Álvarez , J. González‐Gallego , and M.J. Tuñón , “Melatonin Modulates Mitophagy, Innate Immunity and Circadian Clocks in a Model of Viral‐Induced Fulminant Hepatic Failure,” Journal of Cellular and Molecular Medicine 24, no. 13 (2020): 7625–7636, 10.1111/jcmm.15398.32468679 PMC7339179

The above article, published online on 29 May 2020 in Wiley Online Library (http://onlinelibrary.wiley.com/), has been retracted by agreement between the journal Editor‐in‐Chief, Stefan N. Constantinescu; the Foundation for Cellular and Molecular Medicine; and John Wiley & Sons Ltd. Following publication, concerns were raised by third parties regarding duplications in panels C and F in Figure 1B. Additional concerns of duplication and splicing were also raised for Figures 2C and 3B, which were investigated and confirmed by the publisher; additionally, portions of Figure 4 were duplicated from Figure 3 of an earlier article by some of the same authors (González‐Fernández et al. 2018 [https://doi.org/10.3389/fphar.2018.00556]). The authors provided their raw data, but this was not sufficient to resolve the concerns, and the authors were unable to provide a satisfactory explanation. The retraction has been agreed upon because of concerns that portions of the figures were duplicated, affecting the interpretation of the data and results presented.

